# Derivation of hypermethylated pluripotent embryonic stem cells with high potency

**DOI:** 10.1038/cr.2017.134

**Published:** 2017-10-27

**Authors:** Siqin Bao, Walfred WC Tang, Baojiang Wu, Shinseog Kim, Jingyun Li, Lin Li, Toshihiro Kobayashi, Caroline Lee, Yanglin Chen, Mengyi Wei, Shudong Li, Sabine Dietmann, Fuchou Tang, Xihe Li, M Azim Surani

**Affiliations:** 1 The State Key Laboratory of Reproductive Regulation and Breeding of Grassland Livestock, Inner Mongolia University, Hohhot 010070, China; 2 Research Center for Animal Genetic Resources of Mongolia Plateau, College of Life Sciences, Inner Mongolia University, Hohhot 010070, China; 3 Wellcome Trust Cancer Research UK Gurdon Institute, Tennis Court Road, University of Cambridge, Cambridge CB2 1QN, UK; 4 BIOPIC, School of Life Sciences, Peking University, Beijing 100871, China; 5 Cancer Research UK and Medical Research Council Oxford Institute for Radiation Oncology, Department of Oncology, University of Oxford, Oxford OX3 7DQ, UK; 6 Wellcome Trust-Medical Research Council Stem Cell Institute, Tennis Court Road, University of Cambridge, Cambridge CB2 3EG, UK; 7 Inner Mongolia Saikexing Institute of Breeding and Reproductive Biotechnology in Domestic Animal, Hohhot 011517, China; 8 Current address: Center for Genomic Integrity, Institute for Basic Science, Ulsan National Institute of Science and Technology, Ulsan 44689, Republic of Korea

**Keywords:** ESCs, pluripotency, blastocysts, chimeras, yolk sac, placenta, hypermethylated epigenome

## Abstract

Naive hypomethylated embryonic pluripotent stem cells (ESCs) are developmentally closest to the preimplantation epiblast of blastocysts, with the potential to contribute to all embryonic tissues and the germline, excepting the extra-embryonic tissues in chimeric embryos. By contrast, epiblast stem cells (EpiSCs) resembling postimplantation epiblast are relatively more methylated and show a limited potential for chimerism. Here, for the first time, we reveal advanced pluripotent stem cells (ASCs), which are developmentally beyond the pluripotent cells in the inner cell mass but with higher potency than EpiSCs. Accordingly, a single ASC contributes very efficiently to the fetus, germline, yolk sac and the placental labyrinth in chimeras. Since they are developmentally more advanced, ASCs do not contribute to the trophoblast. ASCs were derived from blastocysts in two steps in a chemically defined medium supplemented with Activin A and basic fibroblast growth factor, followed by culturing in ABCL medium containing ActA, BMP4, CHIR99021 and leukemia inhibitory factor. Notably, ASCs exhibit a distinct transcriptome with the expression of both naive pluripotency genes, as well as mesodermal somatic genes; *Eomes, Eras, Tdgf1, Evx1, hand1, Wnt5a* and distinct repetitive elements. Conversion of established ESCs to ASCs is also achievable. Importantly, ASCs exhibit a stable hypermethylated epigenome and mostly intact imprints as compared to the hypomethylated inner cell mass of blastocysts and naive ESCs. Properties of ASCs suggest that they represent cells at an intermediate cellular state between the naive and primed states of pluripotency.

## Introduction

Development of zygotes to blastocysts is accompanied by a sequential change from totipotency to pluripotency, with differentiation of the outer trophectoderm cells and the pluripotent cells in the inner cell mass (ICM). The ICM gives rise to all the somatic tissues, as well as germ cells. Pluripotent embryonic stem cells (ESCs) isolated from the ICM are *in vitro* counterparts^1,2,3^, which when introduced into the blastocysts can differentiate into all fetal tissues and germ cells, albeit making an insignificant contribution to the extraembryonic tissues^4^.

Following blastocyst implantation, epiblast cells in postimplantation embryos retain some characteristics of pluripotency while becoming progressively more hypermethylated before the initiation of somatic cell fates^5,6^. Epiblast stem cells (EpiSCs) from postimplantation epiblast cells are hypermethylated but retain some features of pluripotency^7,8^, where female EpiSCs have an inactive X chromosome^9^. A variety of self-renewing pluripotent stem cells have been reported^10,11,12,13,14^, with variable potencies, epigenetic states and transcriptional regulators. These cells have been generated *in vitro* using empirical combinations of cytokines, fetal calf serum and hormones, including mouse embryonic fibroblasts (MEF) as feeders.

Here, ASCs showed unique properties with the expression of naive pluripotency and some mesodermal somatic genes in chemically defined medium without serum. Importantly, these cells were also hypermethylated relative to naive ESCs. Altogether, ASCs represent an early postimplantation epiblast-like state of development. However, when injected into host blastocysts, single ASC contributed to embryonic tissues, germline, yolk sac and placental labyrinth that originate from the postimplantation epiblast, but did not contribute to the trophoblast. ASCs exhibit unique features and provide a new model for investigations on the epigenetic and transcriptional states that regulate cell potency.

## Results

### Derivation and characterization of AFSCs

**Figure 1:**
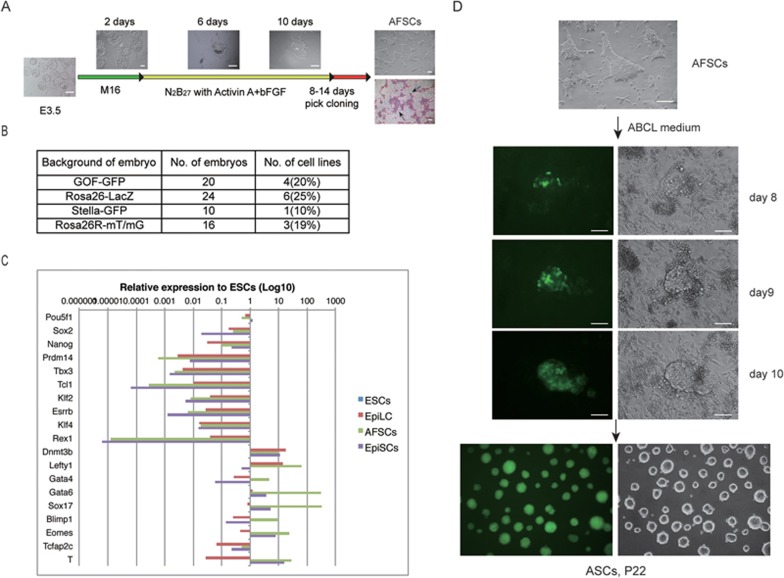
Derivation of AFSCs and ASCs. **(A)** Schematic overview of AFSC derivation. Note AP-positive cells (arrow indicated) in AFSCs. Scale bars, 100 μm. **(B)** Derivation rate of AFSCs with diverse genetic backgrounds. **(C)** Quantitative RT-PCR of key genes in ESCs, EpiLCs, AFSCs and EpiSCs, all derived from 129/sv females mated with GOF-GFP males of mixed background. Note the expression of endoderm and mesoderm genes in AFSCs (at the bottom). **(D)** Reprogramming AFSC to ASCs. Scale bars, 100 μm.

First, we attempted to derive stem cells from blastocysts in a chemically defined medium with N2B27 in the presence of ActA and basic fibroblast growth factor (bFGF) (but excluding KSR and feeder cells). Most of the E3.5 blastocysts attached within 5-6 days, forming ICM outgrowth, of which ∼20% resulted in stable lines from diverse genetic backgrounds; we designated these as ActA/bFGF stem cells (AFSCs) (Figure 1A and 1B). The rate of AFSC derivation was similar to that reported previously for naive ESCs in 2i/LIF^15^.

Immunofluorescence (IF) and RT-qPCR analysis of AFSCs revealed expression of OCT4, SOX2, NANOG, E-cadherin; we also detected H3K27me3 which is a distinctive mark of an inactive X chromosome (Figure 1C; Supplementary information, Figure S1A). AFSCs share similarities with EpiSCs^7,8^, where expression of some genes of the ICM and ESCs were significantly reduced, including *Prdm14, Tbx3, Tcl1, Klf2, Esrrb, Klf4* and *Rex1*. By contrast, we observed higher expression of early germ layer genes *Eomes, Lefty1* and *brachyury* (*T*) (Figure 1C), together with the strong expression of *Sox17* and *Blimp1*. AFSCs also uniquely displayed expression of *Gata6 and Gata4,* which are a part of the primitive endoderm transcriptome (Figure 1C). Overall, AFSCs were clearly at a stage beyond the pluripotent state of epiblast cells in blastocysts but as teratomas, they were still able to differentiate into cells of all the three germ layers (Supplementary information, Figure S1B).

Next, following the injection of 15-20 AFSCs with *Rosa26-LacZ* reporter into E3.5 blastocysts, we detected their contribution (∼11%-14%) in chimeric embryos at E6.5-E8.5 (Supplementary information, Figure S1C, S1D and Table S1A), which is unlike EpiSCs^7,8^. More strikingly, their high contribution was found in the posterior primitive streak and the extra-embryonic mesoderm surrounding the epiblast in E6.5-E7.5 chimeric embryos (Supplementary information, Figure S1C and S1D). The latter develops into the yolk sac and contributes in part to the placenta. Thus, AFSCs are distinct and, unlike pluripotent EpiSCs.

### Derivation and characterization of ASCs

Next, we explored the impact of Wnt and BMP4 signaling on AFSCs, which can substitute for serum in ESC cultures^16,17^, and induce differentiation of EpiSCs^18,19^. Using CHIR99021 (CH) for simulating canonical Wnt signaling^20^, we started with two established AFSCs cell lines with GOF-GFP or Stella-GFP reporters. AFSCs were dissociated and cultured in N2B27 medium with ActA, BMP4, CH and LIF (henceforth called ABCL medium) (Figure 1D). After 10 days, we detected GOF-GFP-positive clones, which were able to self-renew for more than 30 passages. We designated these cells as advanced pluripotent stem cells (ASCs) and compared them with EpiSCs (Supplementary information, Table S1B).

**Figure 2:**
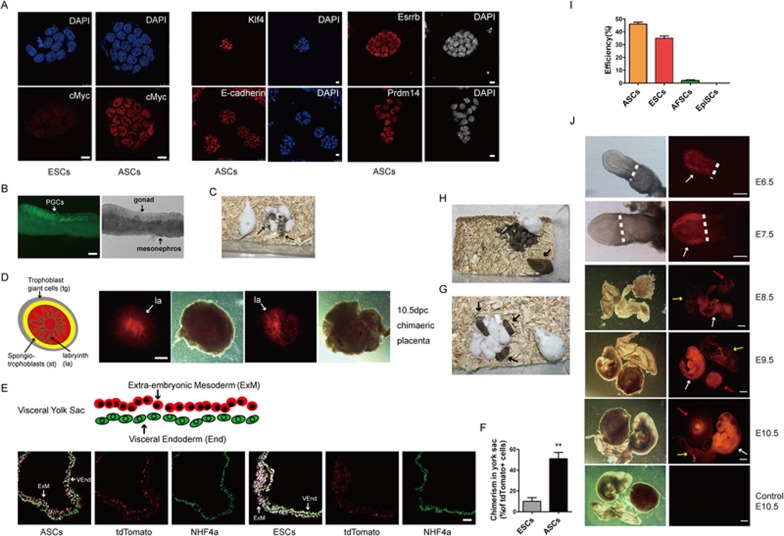
Characteristics of ASCs. **(A)** IF staining assays in ESCs and ASCs, including those detect the expression of KLF4, E-CADHERIN, ESSRB and PRDM14 in ASCs. Scale bars, 10 μm. **(B)** Germline transmission of ASCs in E12.5 chimeras shown by GOF-GFP-positive cells (arrow). Scale bars, 100 μm. **(C)** Chimeric pups (arrows) generated by injecting ASCs in ICR host blastocysts. **(D)** A schematic depiction of placenta tissue indicates the contribution of ASCs (tdTomato) to labyrinth of placenta in E10.5 chimeras (white arrows). Scale bars, 1 mm. **(E**, **F)** Schematic depiction of the yolk sac, and the contribution of ASCs (tdTomato) to the extraembryonic mesoderm (red) in E10.5 chimeras. Scale bars, 100 μm. **(G)** Pups (arrows) generated entirely from ASCs (between 129/sv females and GOF-GFP males) in tetraploid ICR host blastocysts. **(H)** The F1 pups generated by ASCs-tetraploid male (arrows) mated with ICR female. **(I)** The efficiency of clonal line derived from single ASCs. **(J)** Chimeras (E6.5-E10.5) generated with single ASC. White arrows indicate embryonic body; yellow arrows mark yolk sac; and red arrows point placenta. Scale bars for E6.5-7.5, 200 μm; scale bars for E8.5-10.5, 2 mm. ExM, extraembryonic mesoderm; la, labyrinth; st, spongetrophectoderm; tg, trophectoderm giant cell; VEnd, visceral endoderm.

To determine the key features of ASCs relative to ESCs, we examined expression by IF for cMYC, KLF4, E-cadherin, ESRRB and PRDM14. Excluding the higher expression of cMYC in ASCs than in ESCs, expression of other genes was equivalent to that in ESCs (Figure 2A). Notably, high expression of cMYC in ESCs promotes rapid transition through the G1 phase of the cell cycle, suppresses differentiation and controls pluripotency^21^.

Next, we assessed the differentiation potential of ASCs with GOF-GFP reporter in chimeric embryos by injecting 15 cells into individual blastocysts. We found that ASC contributed to the embryo proper and germline of E12.5 embryo, as well as to the full-term pups (Figure 2B and 2C; Supplementary information, Table S1C and S1D). Importantly, ASCs contributed to the placenta in chimeric conceptuses (Supplementary information, Figure S2A), which was also the case with the other ASC line harboring Stella-GFP reporter (Supplementary information, Figure S2B). Furthermore, using ASCs with GOF-GFP and a constitutive tdTomato reporter, we detected these cells in the placental labyrinth (Figure 2D; Supplementary information, Figure S2C), yolk sac (Figure 2E and 2F), embryo proper and the germline at E10.5-E13.5 (Supplementary information, Figure S2D, S2E and Table S1E). Notably, ASCs contributed to more than 50% of the extra-embryonic mesoderm of yolk sac, but there was no detectable contribution to the extra-embryonic endoderm of yolk sac (Figure 2E and 2F). This result indicates that ASCs are in a stable state between pluripotency and the onset of differentiation, with a potential to contribute to embryos, yolk sac and placental labyrinth.

For a further stringent test of the potency of ASCs, we introduced them into tetraploid host blastocysts, where the donor cells contribute predominantly if not exclusively to the embryo^22^. Using 15 donor ASCs, we obtained live pups with normal fertility at a higher rate than reported previously^23,24^ (Figure 2G and 2H; Supplementary information, Table S1F). The derivation efficiency of clonal lines from single ASC was equivalent to that of naive ESCs and much higher than that of EpiSCs or AFSCs (Figure 2I). Thus WNT and BMP4 signaling pathways increase the potency of AFSCs in the course of ASC derivation, and this potency exceeds that shown by EpiSCs *in vivo*.

Finally, we tested the potency of ASCs more stringently by introducing single ASCs into 8-cell stage embryos, which were cultured overnight and then transferred to recipient females. We found that 24%-36% of the embryos at E6.5-E7.5 and 31%-50% of the embryos at E8.5-E10.5 contained derivatives of ASCs with tdTomato reporter (Figure 2J; Supplementary information, Figure S2F and Table S2A). We also observed germline transmission and full-term chimeras from single ASCs (Supplementary information, Table S2B and S2C). Additional analysis of 18 conceptuses at E8.5-E10.5 resulting from single donor ASCs showed 14 chimeras (78%) with contributions to the embryo, yolk sac and the placenta (Figure 2J; Supplementary information, Figure S2F and Table S2A). In contrast, single naive donor ESCs cultured in 2i/LIF resulted in just 2/20 chimeras, with a contribution to the embryo, and no contribution to the extraembryonic tissues. When the number of donor cells was increased to 15, we obtained 18 chimeras, in which donor cell contributed predominantly to the fetus. However, in only one of these chimeras, ESC cells contributed to the fetus, yolk sac and the placenta (Supplementary information, Table S2D). Altogether, ASCs exhibit a unique pluripotent state, with high clonogenicity and potency, and a single ASC contributed to the embryo, placental labyrinth and the germline.

To test for the potential for the conversion of ESCs (2i/LIF) to ASCs, we cultured two ESC cell lines (SQ3.3 and E14). Both cell lines demonstrated normal growth and were passaged over 20 times in ABCL medium; and the resulting cells was designated as ESC-ASCs. We tested the *in vivo* developmental potential of ESC-ASCs in chimeras using single or multiple ESC-ASCs. We found that both single and multiple ESC-ASCs contributed to the embryo, yolk sac and placenta labyrinth (Supplementary information, Figure S2G; Supplementary information, Table S2E). Thus ESCs can be converted to ASCs by culturing in ABCL, exhibiting increased potency and characteristics resembling ASCs.

### Molecular features of ASCs

**Figure 3:**
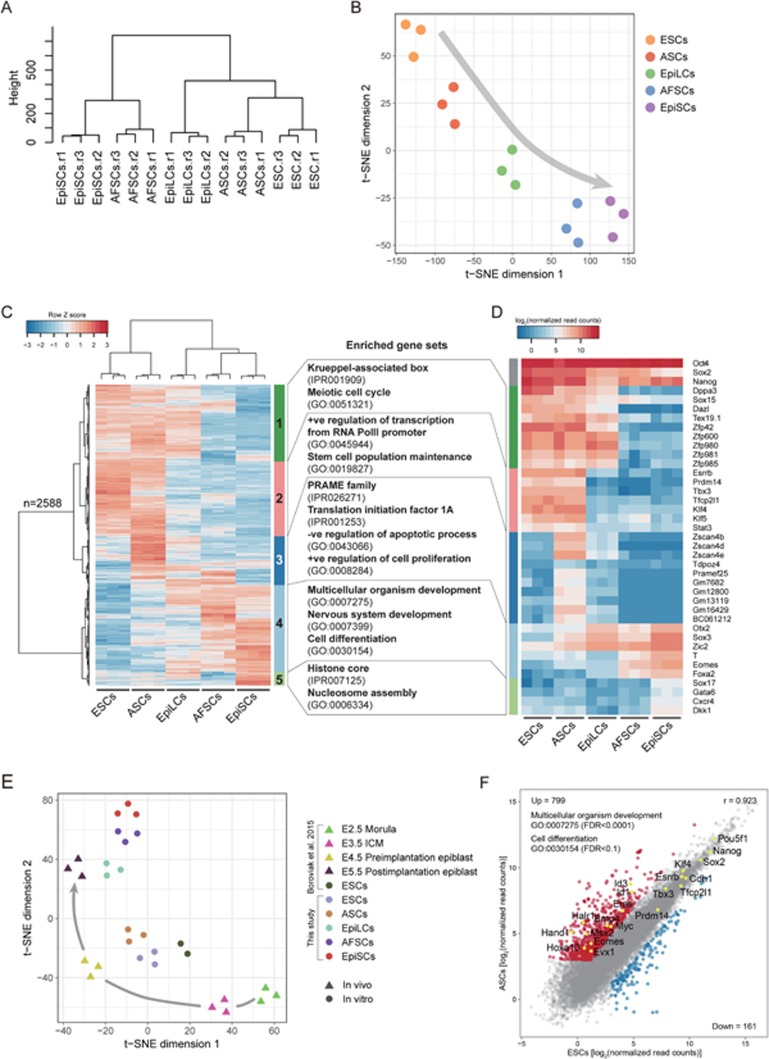
RNA-seq analysis of AFSCs and ASCs. **(A)** Unsupervised hierarchical clustering (UHC) of whole-genome transcriptome from three biological replicates. Note that ASCs were clustered close to ESCs but not EpiSCs. **(B)** t-distributed stochastic neighbor embedding (t-SNE) of whole-genome transcriptome. Arrow represents potential trajectory from naive to primed pluripotent stem cells. **(C)** Heatmap showing scaled expression values of 2 588 differentially expressed genes (mean log_2_(normalized read counts) > 3 in any sample, log_2_(fold change) > 3, adjusted *P*-value < 0.05) between the five cell types. UHC of five major gene clusters. The top representative GO biological process and InterPro terms for each cluster are indicated on the right. **(D)** Expression heatmap of representative genes for each cluster in **C**. **(E)** t-SNE analysis of gene expression of pluripotent stem cells, and of E2.5-E5.5 embryos^25^, based on 1 685 dynamically expressed genes^25^. Arrow indicates developmental progression from E2.5 morula to E5.5 postimplantation epiblast. **(F)** Pair-wise gene expression comparison between ASCs and ESCs (2i/LIF). Upregulated (red) and downregulated (blue) genes in ASCs were highlighted (mean log_2_(normalized read counts) > 3 in either samples, log_2_(fold change) > 2, adjusted *P*-value < 0.05). Gene ontology analysis (by DAVID) showed ASCs upregulated genes that are involved in multicellular organism development and cell differentiation.

To investigate the molecular features of ASCs, we profiled their transcriptome by RNA sequencing (RNA-seq), and compared it with naive ESCs (cultured in 2i/LIF), EpiSCs and EpiLCs (epiblast-like cells) (Supplementary information, Table S3A). Unsupervised hierarchical clustering (UHC) showed that ASCs and naive ESCs cluster together, whereas AFSCs are close to EpiSCs (Figure 3A; Supplementary information, Figure S3A). Moreover, the t-distributed stochastic neighbor embedding (t-SNE) analysis indicated a trajectory representing naive to primed cell states from ESCs and ASCs toward EpiLCs, and to more advanced AFSCs and EpiSCs (Figure 3B).

We examined the similarities and differences among the five cell types by analyzing a total of 2 588 differentially expressed genes. These genes were segregated into five distinct classes by UHC (Figure 3C; Supplementary information, Table S3B and S3C). All the five pluripotent cell types expressed core pluripotency factors *Oct4, Sox2* and *Nanog* (Figure 3D), but the expression of naive pluripotency genes (e.g., *Esrrb*, *Tfcp2l1*, *Klf4*, *Tbx3* and *Prdm14*) was detected exclusively in ASCs and ESCs (cluster 2, Figure 3C and 3D), which was confirmed by IF analysis (Figure 2A). On the other hand, AFSCs and EpiSCs showed enrichment for developmental genes, including *Otx2*, *Zic2*, *T*, *Eomes* and *Foxa2* (cluster 4, Figure 3D).

Notably, we found that 421 genes were exclusively expressed in ASCs (cluster 3, Figure 3C), including genes that encode preferentially expressed antigen of melanoma (PRAME) family and translation initiation factor 1A (see below). Principal component analysis (PCA) of transposable element expression also showed a similar trajectory from naive to primed cell states as seen by analyzing the overall gene expression profiles (Supplementary information, Figure S3B). Notably, both ESCs and ASCs showed high expression of *RLTR45*, *RMER16* and *LTRIS2* (Supplementary information, Figure S3C), which might distinguish them from EpiSCs.

Next, we tested if ASCs correspond to cells at a particular embryonic developmental stage by comparing the ASC transcriptome with that of E2.5-E5.5 embryos^25^ (Supplementary information, Table S3D). We performed t-SNE and UHC analyses with 1 685 genes that are dynamically expressed between E2.5 and E5.5 stages^25^. Naive ESCs and ASCs were clustered with E4.5 preimplantation epiblast, with ASCs being slightly more advanced than ESCs (Figure 3E; Supplementary information, Figure S3D). EpiLCs were clustered with E5.5 postimplantation epiblast, while AFSCs and EpiSCs had progressed to more advanced stages^26,27,28^ (Figure 3E; Supplementary information, Figure S3D). Consistently, the comparison between ASCs and naive ESCs suggested the expression of the core genes (e.g., *Oct4*, *Nanog* and *Sox2*) and naive pluripotency genes (e.g., *Esrrb*, *Klf4* and *Tfcp2l1*) in both of them. However, ASCs also showed upregulation of some primitive streak and mesoderm genes, including *Eomes*, *Eras, Evx1, Hand1* and *Hoxa10* (Figure 3F; Supplementary information, Figure S3E). Furthermore, ASCs exhibited high gene expression level and protein abundance for cell-adhesion molecule E-cadherin (Cdh1) and c-Myc (Figures 2A and 3F). Although T and Cdx1 IF also showed heterogeneity among ASCs, the consistent contribution of single ASCs to chimeras suggests the presence of a dynamic equilibrium (Supplementary information, Figure S3F). We also noted a slightly higher expression of *Hoxa* genes in ASCs compared to ESCs, including *Hoxa1, Hoxa5, Hoxa9, Hoxa10, Hoxa11, Hoxa11os*, and *Hoxa13* (Supplementary information, Figure S3G). In this context, the lncRNA *Haunt/Halr1* that locates ∼40 kb upstream of the *Hoxa* cluster^29,30,31^ also displayed higher expression in ASCs than ESCs (Supplementary information, Figure S3G).

Further examination of ASCs (cluster 3, Figure 3C; Supplementary information, Table S3B) revealed expression of so-called 2C genes: *Zscan4b*, *Zscan4d* and *Zscan4e* (Figure 3D; Supplementary information, Figure S4A and Table S3E), and *MERVL* transcripts (Supplementary information, Figure S3C)^12,32,33,34^. However, IF analysis showed that only 8% of the ASCs were MERVL-Gag positive/OCT4 negative, but such cells were rarer in 2i/LIF ESCs (Supplementary information, Figure S4A and S4B). Nonetheless, this does not account for the potency of ASC, since ESC-ASCs showed similar characteristics in the absence of 2C-like gene expression (Supplementary information, Figures S2G, S4C and S4D).

To this end, we injected a single ASC cell with tdTomato and GOF-GFP into an 8-cell stage embryo and examined its chimeric contribution after 48 h of *in vitro* culturing. Consistently, ASC contributed only to the ICM, and not to the trophoblast in blastocysts (0/42 recovered blastocysts) (Supplementary information, Figure S4E).

### ASCs exhibit a hypermethylated stable methylome

Naive ESC pluripotent state displays global DNA hypomethylation, where both X chromosomes are active in female cells^15,35,36^. Considering the enhanced potency of ASCs described above, we examined the epigenome of ASCs. Our initial IF observations showed bright H3K27me3 foci in female AFSCs, but not in female ASCs, suggesting reactivation of the X-chromosome in ASCs (Supplementary information, Figure S5A).

**Figure 4:**
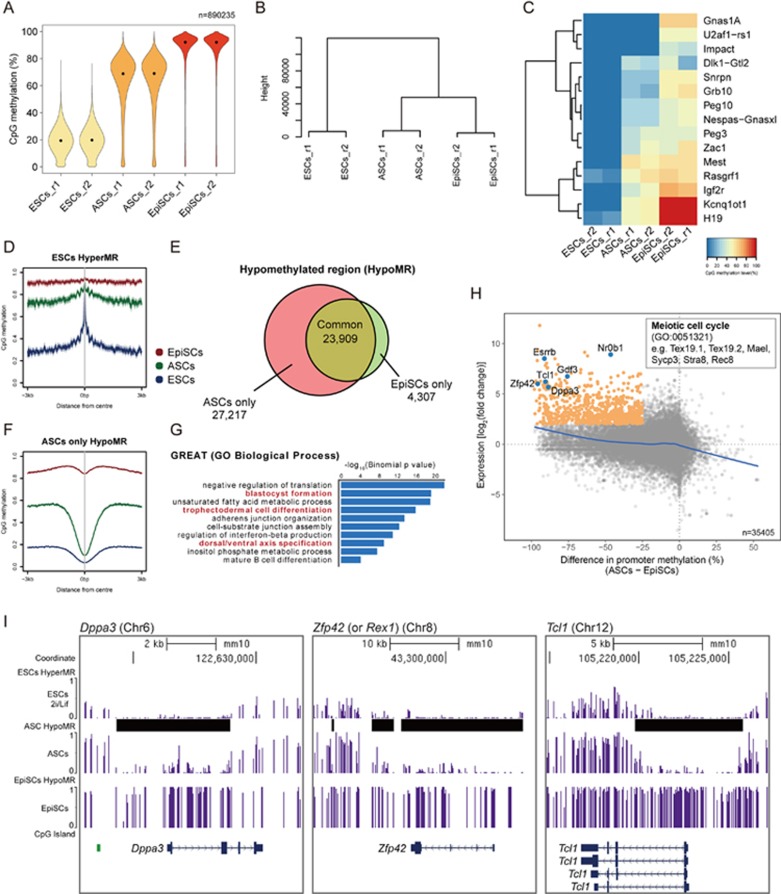
Methylation analysis of ASCs. **(A)** Violin plot showing CpG methylation distribution of 2 kilobase (kb) genomic tiles. **(B)** UHC of methylation levels of 2 kb genomic tiles. **(C)** Heatmap showing CpG methylation of imprint control regions (ICRs) in ESCs, ASCs and EpiSCs. **(D)** Methylation profiles of ESCs hypermethylated regions (HyperMR) in ESCs. Note that HyperMR in ESCs were generally hypermethylated in ASCs and EpiSCs. **(E)** Venn diagram showing overlap of hypomethylated regions (HypoMR) between ASCs and EpiSCs. **(F)** Methylation profile of ASC-specific HypoMRs in **E**. **(G)** GREAT analysis of ASC-specific HypoMRs. **(H)** Scatterplot of differential gene expression and difference in promoter methylation between ASCs and EpiSCs. Genes with > 20% promoter methylation and log_2_ (read counts) > 3 in either samples were shown. Genes upregulated in ASCs with promoter demethylation are highlighted in orange. They were enriched for regulating meiotic cell cycle and cell pluripotency. Best fit curve (blue line) was generated by the generalized additive models (GAM). **(I)** UCSC genome browser snapshots of CpG methylation level at representative naive pluripotency genes.

We further performed DNA methylation analysis of female ASCs, ESCs and EpiSCs by whole-genome bisulfite sequencing (BS-seq) (Supplementary information, Table S3F). Although naive ESCs (2i/LIF) were globally hypomethylated (median CpG methylation of ∼20%), ASCs showed a hypermethylated genome (median ∼70%) (Figure 4A). Indeed, UHC analysis revealed that the methylome of ASCs was more similar to the hypermethylated epigenome of EpiSCs (median 90%) (Figure 4B; Supplementary information, Figure S5B). Most genomic regions, including promoters, enhancers, exons, introns and intergenic regions, were highly methylated in ASCs (Supplementary information, Figure S5C). In line with a previous report^35^, all genomic imprints were erased in female 2i/LIF ESCs, whereas most imprints were retained in both ASCs and EpiSCs (Figure 4C). The retention of imprints, which are essential for development, would make ASCs more robust in culture, and during subsequent differentiation.

To characterize the methylome further, we calculated hypermethylated regions (HyperMRs) in ESCs and hypomethylated regions (HypoMRs) in ASCs and EpiSCs using a hidden Markov model^37^. We found very few HyperMRs in 2i/LIF ESCs (1 910); and most of these regions also showed high methylation levels in ASCs and EpiSCs (Figure 4D). We then focused on comparing the methylomes of ASCs and EpiSCs. Among the 51 126 HypoMRs in ASCs, 27 217 (53%) were not present in EpiSCs (Figure 4E and 4F; Supplementary information, Table S3G). Analysis using Genomic Regions Enrichment of Annotations Tool (GREAT)^38^ suggested that the ASC-specific HypoMRs (CpG coverage > 40, methylation difference > 40%) were in the vicinity of genes that function in blastocyst formation (e.g., *Esrrb* and *Oct4*), trophectodermal cell differentiation (e.g., *Tead4*, *Tfap2c*, *Eomes*) and dorsal/ventral axis specification (e.g., *Wnt3*, *Pax6*) (Figure 4G). Promoter methylation and gene expression analysis between ASCs and EpiSCs suggested an overall weak anti-correlation (Figure 4H). However, we found that a small fraction of genes (551) were upregulated in ASCs and exhibited promoter demethylation. They were enriched for genes involved in regulating Meiotic cell cycle (e.g., *Mael*, *Sycp3* and *Stra8*) (Figure 4H; Supplementary information, Table S3H and S3I). These germline genes are promoter methylation-sensitive and are expressed in naive ESCs, where their promoters are demethylated. In addition, we found that naive pluripotency genes, such as *Dppa3, Zfp42* (also known as *Rex1*), *Tcl1, Esrrb, Gdf3 and Fbxo15*, were also promoter demethylated and upregulated in ASCs (Figure 4H and 4I; Supplementary information, Figure S5D). Thus, ASCs exhibited an overall hypermethylated genome and maintained imprints, but showed targeted demethylation at loci associated with the preimplantation development and naive pluripotency. This is consistent with their high contribution in chimeras described above.

## Discussion

We describe the derivation of two novel cell types, AFSCs and ASCs from mouse blastocysts, which provide new insights on pluripotent stem cells. In particular, the ASCs (advanced pluripotent stem cells) show higher DNA methylation levels than naive ESCs (Figure 4A and 4B), and with the expression of both pluripotent and somatic genes (Figure 3C; Supplementary information, Figure S3E). We also find that genomic imprints are more stable in ASCs relative to naive ESCs (Figure 4C). On the basis of the transcriptome and DNA methylome analysis, ASCs appeared to be at an intermediate state between naive ESCs and primed EpiSCs, and apparently represent the cells with the characteristics of the early postimplantation epiblast. Remarkably, ASC has high developmental potency in chimeras, since a single ASC contributes extensively to the developing embryo, germline and the extraembryonic mesoderm (Figure 2J). Indeed it is possible to obtain a live pup consisting exclusively of ASCs in “tetraploid rescue experiments”. The high potency of individual ASCs may in part be due to the shorter cell cycle time (data not shown) and higher expression of cMYC in ASCs as compared to that in ESCs (Figures 2A and 3F). Accordingly, cMYC might promote rapid cell transition through the G1 phase of the cell cycle while suppressing cell differentiation by activating Dusp2 and Dusp7 phosphatases, which in turn represses FGF/ERK signaling^21^. The relevance and significance of the cMyc-Dusp2-FGF/ERK pathway in ASCs merit further investigation.

Concerning the extraembryonic tissues, individual ASCs do not contribute to the trophectoderm cells when introduced into 8-cell stage embryos (Supplementary information, Figure S4E), unlike some recently described pluripotent stem cells^11,12,13,14^. This observation is consistent with ASCs being equivalent to early postimplantation epiblast cells, and therefore developmentally they represent cells at a later stage when we would not expect their contribution to the trophoblast cells, which also accounts for our observation that ASCs did not contribute to the extraembryonic endoderm. Nevertheless, single ASCs contribute significantly to some extraembryonic tissues that originate from the postimplantation epiblast, especially the yolk sac mesoderm and placental labyrinth in chimeric conceptuses as compared to ESCs; this is also a mark of higher potency of ASC^39^. Altogether, ASC contribute extensively to the tissues that normally originate from postimplantation epiblast cells, which is consistent with their overall molecular state and characteristics.

Another feature of interest concerning ASC involves the *Hoxa* family genes. We found that the lncRNA *Haunt/Halr1* located ∼40 kb upstream of the *Hoxa* cluster and showed higher expression in ASCs than in ESCs (Supplementary information, Figure S3G). The *Huant/Halr1* genomic locus has been shown to be bound by pluripotency regulators, including *Oct4, Nanog and Sox2*, with enhancer function-related epigenetic marks, such as H3K4me1, DNase I hypersensitivity, H3K4me3 and H3K36me3^29,30^. The *Haunt* lncRNA and its genomic locus regulate the *Hoxa* gene cluster during differentiation of pluripotent cells^31^. Our data suggest that high expression of *Haunt/Halr1* in ASCs may promote downregulation of Hoxa cluster to maintain a stable pluripotent state.

How precisely the chemically defined ABCL medium induces and maintains the hypermethylated state in ASC deserves further investigation in the future. The relatively hypermethylated state of ASCs with intact genomic imprints compared to naive ESCs is likely to provide greater stability during self-renewal *in vitro*, which is consistent with the findings of two recent studies on ESCs^40,41^.

The AFSCs we describe in this study are quite similar to EpiSCs, which differentiate in the presence of Wnt and BMP4^18,19^. On the other hand, Activin A and LIF support self-renewal of AFSCs and ESC^17^, respectively. Thus, during the derivation of ASCs, BMP4 and CH would likely promote AFSCs toward differentiation, which would be counteracted by Act A and LIF that supports self-renewal. Under these signaling conditions, a small proportion of surviving cells acquire new phenotypes and we describe these cells as ASCs. Further detailed analyses, including analysis of epigenetic resetting, determining how precisely the ASCs become established in culture, need to be explored in the future studies. Notably, ESCs can also be readily converted to ASCs, referred to here as ESC-ASCs, with similar properties.

In conclusion, ASCs are a novel and distinct self-renewing type of pluripotent stem cells, which exhibit intermediate features between ESCs and EpiSCs. Transcriptionally, ASCs are at a cellular state that beyond the epiblast state of the blastocyst. In this respect, ASCs are like EpiSCs, albeit some loci remain hypomethylated in ASCs, and importantly, female ASCs do not exhibit X-inactivation. Thus, ASC represents a unique pluripotent state with distinct transcriptional and epigenetic features, which confers greater stability and potency.

## Materials and Methods

### Derivation of AFSC cell lines

Mouse embryos at E3.5 were isolated from 129/sv females mated with Oct4-ΔPE-GFP (GOF-GFP), Stella-GFP, Rosa129/Sv and Rosa26R-mT/mG transgenic males with a mixed background of MF1, 129/sv and C57BL/6J strains^42,43,44^. The GOF-GFP expression of the reporter that lacked the proximal enhancer and was under the control of the Oct4 promoter and distal enhancer. This GFP transgene showed expression in the ICM of blastocysts, PGC *in vivo* and in ESC. Rosa129 is a knockin mouse line with a LacZ reporter in the Rosa locus, where LacZ is ubiquitously expressed. Standard AF medium consists of N2B27 medium (Life technology) supplemented with Activin A (20 ng/ml, R&D Systems) and bFGF (12 ng/ml, R&D systems). E3.5 blastocysts were cultured in M16 medium (Sigma-Aldrich) for 2 days. Most of them were zone pellucida free. If blastocysts had zona pellucida, they were removed by Acidic Tyrode's Solution (Sigma-Aldrich). They were then placed in 24-well fibronectin-coated (16.7 μg/ml, Millipore) plate with AF medium. Epiblast cultures grew efficiently and formed “flat” epithelial-like colonies after 5-6 days. The resulting colonies were further cut into smaller pieces by glass needles after 7-9 days of culturing, and then the colonies passaged by Accutase (Life technology) regularly every 2-3 days. These cells, referred to as AFSCs, could be passaged for over 30 passages in AF medium.

### Derivation of ASC cell lines

We dissociated GOF-GFP AFSCs using Accutase and placed AFSCs (3 × 10^5^ single cells) in fibronectin-coated 24-well plates with 1 ml ABCL medium. The ABCL medium was N2B27 medium with Activin A (20 ng/ml, R&D systems), BMP4 (50 ng/ml, R&D systems), CHIR9902 (3 μM, Miltenyi Biotech) and leukemia inhibitory factor (1 000 IU/ml, Millipore). Dependent on cell growth, AFSCs were then passaged after 2-4 days. After 6-7 days of ABCL treatment, we noted cell dying. GOF-GFP-positive clones started to appear around day 10-14. When these GFP-positive colonies grew to around 200 μm in diameter, they were cut into smaller pieces using glass needles and then transferred. When these colonies had grown for 6-7 days, they were treated with Accutase, and the resulting cells were cultured to produce GFP-positive colonies, which were capable of self-renewal for over 30 passages. We called these cells ASCs.

### Teratoma formation

The AFSCs were disaggregated using Accutase, and 1 × 10^6^ cells were injected under the epithelium of NOD-SCID mice. Three to five weeks after transplantation, tumor(s) were collected and fixed with 4% paraformaldehyde, and processed for paraffin sectioning. Sections were observed following hematoxylin and eosin staining.

### Production of chimeras

Approximately, 15-20 cells were injected gently into ICR mice blastocoel cavity of blastocysts (E3.5) using a piezo-assisted micromanipulator attached to an inverted microscope. The injected embryos were cultured in KSOM medium (Millipore) to enable re-expansion of the blastocoel cavity and then transferred to the uteri of pseudopregnant ICR mice at 2.5 days post coitus (dpc). In a similar manner to the injection of blastocysts, 8-cell embryos (E2.5) were injected with a single cell placed carefully into the perivitelline space under the zona pellucida. The embryos were cultured overnight in KSOM medium (Millipore) at 37 °C in a 5% CO_2_ atmosphere and transferred to the uteri of pseudopregnant ICR mice at 2.5 dpc. The embryos were isolated at embryonic stage E6.5-E13.5 with chimeric contribution checked. Full-term chimeras were confirmed by the coat color pattern of the pups at birth.

### Production of full-term pups in tetraploid host blastocysts

Two-cell stage embryos (E1.5) from ICR mice were collected by flushing oviducts; they were subjected to electrofusion to create tetraploid (4N) host blastocysts. Typically 15-20 ASCs were injected into tetraploid host blastocyts, which were transferred to pseudopregnant recipients at 2.5 dpc.

### Immunofluorescence

Cells were briefly washed with PBS and fixed in 4% paraformaldehyde in PBS for 15 min at room temperature. Cells were permeabilized for 30 min with 1% BSA and 0.1% Triton X-100 in PBS. Antibody staining was carried out in the same buffer at 4 °C overnight. The slides were subsequently washed three times in PBS with 1% BSA, 0.1% Triton X-100 (5 min each wash), then incubated with secondary antibody for 1 h at room temperature in the dark, washed once for 5 min in 1% BSA, 0.1% Triton X-100 in PBS and twice for 5 min in PBS. The slides were then mounted in Vectashield with DAPI (Vector Laboratories) and imaged using a BioRad Radiance 2100 confocal microscope. Primary antibodies used were: mouse monoclonal Oct4 (BD Biosciences, 1:200), rat monoclonal Nanog (eBioscience, 1:500), goat polyclonal Sox2 (Santa Cruz, 1:200), rabbit polyclonal H3K27me3 (Upstate, 1:500), rat monoclonal E-cadherin (Takara, 1:40), Klf4 (Abcam, 1:300), cMyc (Abcam, 1:200), Esrrb (Abcam, 1:200), Prdm14 (Abcam, 1:200), HNF4a (Santa Cruz, 1:100). All secondary antibodies used were Alexa Fluor highly cross-adsorbed (Molecular Probes).

### RNA-seq library preparation

The RNA-seq libraries for this paper were constructed by using NEBNext Ultra Directional RNA Library Prep Kit from Illumina. After isolation of total RNA from bulk amount of mouse stem cells using RNeasy Micro Kit (Qiagen), ∼500 ng total RNA was used to enrich mRNA with polyA tails by using NEBNext Magnetic Oligo d(T)25 beads. After RNA fragmentation and random priming, the first strand cDNAs and second strand cDNAs were synthesized, which was followed by end repair, dA-tailing, adapter ligation and PCR enrichment. The RNA-seq libraries were sequenced on HiSeq4000 platform.

### RNA-seq analysis

Paired-end RNA-seq reads were mapped to the mouse reference genome (GRCm38/mm10) with *TopHat2* (https://ccb.jhu.edu/software/tophat), and one mismatch was allowed. The alignments were guided by Ensembl gene models (*Ensembl* release 86). Read counts for either the full transcript or only the first exon were obtained with *featureCounts* (http://bioinf.wehi.edu.au/featureCounts). Transcript or first exon counts were normalized, and the statistical significance of differential expression between samples was assessed using the R Bioconductor *DESeq2* package (https://bioconductor.org/packages/release/bioc/html/DESeq2.html). Transcript and exon counts normalized by *DESeq2* size factors were subsequently normalized by their length/1 000. RNA-seq data^25^ from early mouse embryos were obtained from the European Nucleotide Archive (accession number: ERP007120), and identically processed.

Paired-end reads were further aligned to the mouse reference genome (GRCm38/mm10) with *bowtie* (options: “-M 1 – v2 – best – strata”). RepeatMasker annotations of transposable elements on the mouse reference genome were obtained from the UCSC genome browser table (GRCm38/mm10). Counts for all TE copies and Ensembl transcripts were obtained using *featureCounts*, and TE counts were subsequently processed by DESeq2 as described above, with the exception that TE counts were normalized by the total number of transcript counts instead of *DESeq2* size factors.

The top 90% expressed genes (log_2_(normalized counts)) in the whole RNA-seq data set were used for clustering analysis. Pearson correlation analysis was performed by the R *cor* function with “1-Pearson correlation coefficient” as a distance metric. UHC analysis was performed by the R *hclust* function with the “ward” method and Euclidean distance metric. t-SNE analysis was performed using the R *Rtsne* function omitting an initial PCA step. PCA analysis was performed with the R *prcomp* function on centered and scaled log_2_(normalized counts) expression values. GSEA analysis was performed by the javaGSEA programme developed by the Broad Institute.

### Real-time PCR

The cDNAs from bulk amount (2 μg) of total RNAs were diluted 10 to 40-fold. Then 2 μl of diluted cDNAs was used for each 20 μl real-time PCR reaction (1× Syber Green PCR Master Mix, 0.2 μM of each primer). All reactions were duplicated. The PCR was carried out as follows: first, 95 °C for 10 min to activate the Taq polymerase, followed by 40 cycles at 95 °C for 15 s (for denaturation) and for 1 min at 60 °C for annealing and extension. Finally a dissociation step was run to exclude the possibility of non-specific amplification.

### LacZ staining

The embryos were isolated and washed twice in PBS. Then they were transferred to a cold solution (2% formaldehyde, 0.2% glutaraldehyde, 0.02% NP-40, 1 mM MgCl_2_, 0.01% sodium deoxycholate in PBS) and fixed for 2 h at 4 °C on a rocking platform. The embryos were washed three times in PBS for 2 min each, and stained in lacZ staining solution (0.4 mg/ml Xgal, 4 mM potassium ferrocyanide, 4 mM potassium ferricyanide, 1 mM MgCl_2_, 0.02% NP-40 in PBS) at 30 °C for 18-36 h in dark. Details of ingredient information are shown below:

Formaldehyde, Sigma-Aldrich; Glutaraldehyde, BioChemika (Fluka); NP-40, Roche;

MgCl_2_, Sigma-Aldrich; Sodium deoxycholate, Sigma-Aldrich; Xgal, Melford, MB1001;

Potassium ferrocyanide, Sigma-Aldrich; Potassium ferricyanide, Sigma-Aldrich.

### Alkaline phosphatase staining

Alkaline phosphatase (AP) staining was carried out using AP Staining Kit from Sigma (86R-1KT) according to manufacturer's instructions. Briefly, the cells were fixed by 4% paraformaldehyde for 10 min, and then were stained overnight by AP staining solution at room temperature in the dark.

### BS-seq library preparation

Genomic DNA (two biological replicates per condition) was extracted by QIAamp DNA Micro Kit (Qiagen). Subsequently, 100 ng of genomic DNA was subjected to 3.5 h bisulfite treatment using the Methylcode Bisulfite Conversion Kit (Invitrogen). To monitor the bisulfite conversion efficiency, unmethylated Lambda DNA (0.5 ng, Promega) was spiked-in before conversion. Single-stranded BS-converted DNA was subjected to the post-bisulfite adaptor tagging (PBAT) protocol as described previously^45,46^. Libraries were subjected to single-end 100 bp sequencing on HiSeq 4000 sequencing system (Illumina). Coverage information was summarized in Supplementary information, Table S3.

### BS-seq analysis

PBAT reads were quality-trimmed with *Trim Galore* (http://www.bioinformatics.babraham.ac.uk/projects/trim_galore), and 4 nt random primer sequences at the 5′ end and 1 nt at the 3′ end of all reads were removed. PBAT reads were then mapped to the computationally bisulfite-converted mouse reference genome (GRCm38/mm10) by using *Bismark*^47^ (version: 0.16.3; parameter settings: “*bismark --pbat -N 1 -L 40”*). Potential PCR duplicates were removed using *the deduplicate bismark* command.

Post-alignment analysis was performed by using the *MethPipe* package^37^. First, aligned sequences were converted to *MethPipe* format using the *to-mr* command. Methylation levels and coverage for each symmetric CpG site were calculated by the *methcounts* and *symmetric-cpgs* commands. Average CpG methylation levels of annotated genomic regions, i.e., promoters, exons, repeats, imprint control regions, CGIs, enhancers and 2 kb genomic tiles, were calculated with the *MethPipe roimethstat* program. Only high-confidence genomic regions with at least 40 CpG observations from reads in the region were used in further analyses.

About 2 kb tiles were calculated for all chromosomes with a 1 kb offset. Coordinates of promoters (−1 000 bp and +100 bp from the transcription start site (TSS)), exons, introns and intergenic regions were retrieved from Ensembl (Mouse Release 86). CGI annotations were obtained from the UCSC Table Browser. Imprint control region annotations were obtained from Tomizawa *et al*^48^. Tissue-specific enhancer coordinates (± 1 000 bp from peak; mm9 coordinates were converted to mm10 by the UCSC liftOver tool) were retrieved from the Mouse Encode Project^49^. HyperMRs and hypomethylated regions were calculated by the *hmr* command from the *MethPipe* package.

UHC of 2 kb tile methylation was performed using the *R hclust* command with the “ward” method. Correlation matrix was calculated by the *R corrplot* package. Profile plot was created by the *R* seqplots package. Gene ontology analysis was performed by GREAT^50^ and DAVID Bioinformatics Resource 6.8^50^.

### Data accession

All the sequencing data were deposited in the NCBI, Gene Expression Omnibus (GEO) under accession number GSE99494.

## Author Contributions

SB, XL and MAS designed the research project, prepared and approved the manuscript; WWCT performed whole-genome bisulfite sequencing experiment and analyzed RNA-seq and BS-seq data; SB, BW and SK derived ASCs and analyzed molecular properties; SB, BW performed *in vivo* embryo experiment; JL, LL and FT prepared RNA-seq library; SD contributed to RNA-seq and BS-seq analysis; SL performed clonal lines experiments from single cell and helped proof the manuscript; KT, CL, YC and MW provided technical assistance.

## Competing Financial Interests

The authors declare no competing financial interests.

## Supplementary Information

Supplementary information, Figure S1 Features of AFSCs.

Supplementary information, Figure S2 Chimeras produced by ASCs.

Supplementary information, Figure S3 RNAseq analysis of AFSCs and ASCs.

Supplementary information, Figure S4 2-cell genes expression in ASCs.

Supplementary information, Figure S5 Methylation landscape of ASCs.

Supplementary information, Table S1 The contribution of AFSCs and ASCs in chimera and tetraploid.

Supplementary information, Table S2 The summary of contribution of ASCs and ESC-ASCs in chimeras.
