# Supplementary material for: Derivation of hypermethylated pluripotent embryonic stem cells with high potency
Source: Cell Res. 2017 Oct 27;28(1):22–34. doi: 10.1038/cr.2017.134 (PMC5752839; doi:10.1038/cr.2017.134)
Supplement: Supplementary information, Figure S1 — Features of AFSCs. [file cr2017134x1.pdf]

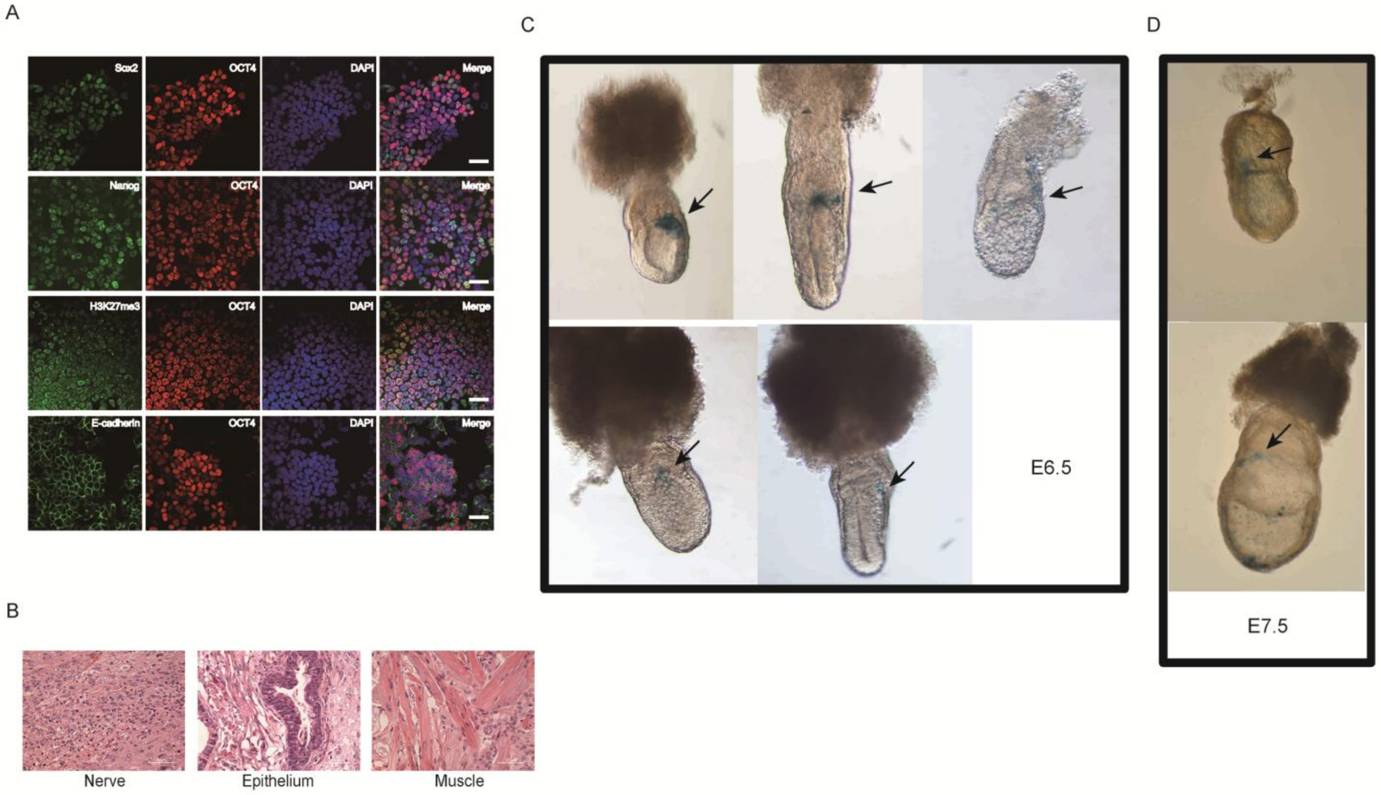

Supplementary information Figure 1 Features of AFSCs.

(A) Immunofluorescence (IF) staining for OCT4, SOX2, NANOG, H3K27me3, and E-Cadherin in AFSCs. Scale bar, 50 μm. **(B)** Differentiation of AFSCs into the three germ layers. Left panel shows a nerve-like structure (Nerve); center panel shows an epithelium-like structure (Epithelium); and right panel shows a muscle–like structure (Muscle). **(C)** E6.5 chimeras derived with Rosa26-lacZ reporter AFSCs. Arrow indicates AFSCs contributed to the primitive streak and part of the extra-embryonic mesoderm. **(D)** E7.5 chimeras with Rosa26-lacZ reporter AFSCs. Arrow shows AFSCs contributed to extra-embryonic tissues.
