# Supplementary material for: Derivation of hypermethylated pluripotent embryonic stem cells with high potency
Source: Cell Res. 2017 Oct 27;28(1):22–34. doi: 10.1038/cr.2017.134 (PMC5752839; doi:10.1038/cr.2017.134)
Supplement: Supplementary information, Figure S2 — Chimeras produced by ASCs. [file cr2017134x2.pdf]

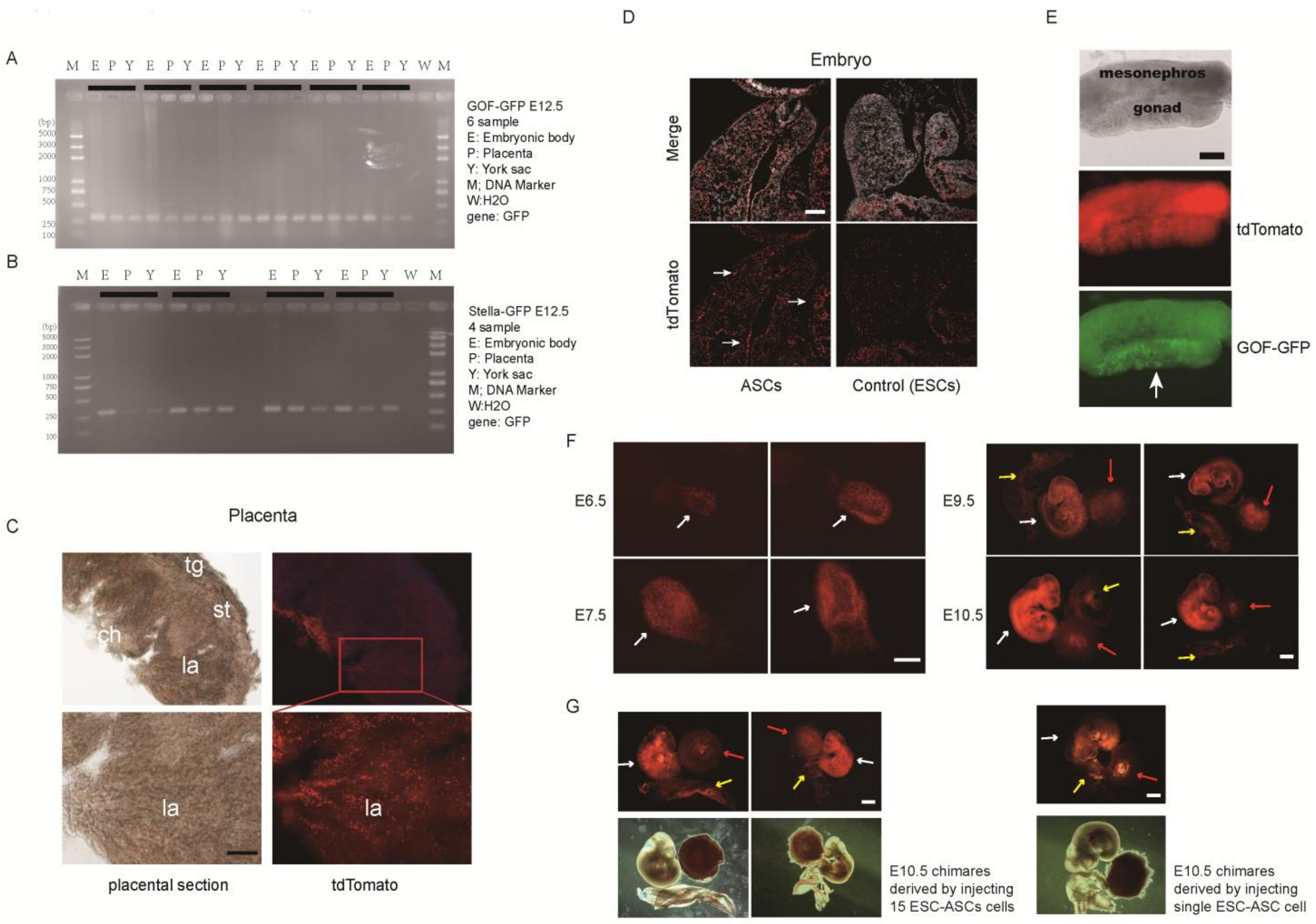

Supplementary information Figure 2 Chimeras produced by ASCs.

(A) ASCs contributed to embryonic body, placenta, and yolk sac in E12.5 GOF-GFP chimeras, detected by genomic PCR. **(B)** ASCs contributed to embryonic body, placenta, and yolk sac in E12.5 Stella-GFP chimeras, detected by genomic PCR. **(C)** ASCs (tdTomato) contributed to placenta in E10.5 chimeras. ASCs exist in labyrinth of placenta. tg, trophectoderm giant cell; st, spongetrophectoderm; la, labyrinth; ch, chorion. Scale bar, 100 μm. **(D)** ASCs (tdTomato) contributed to the embryonic body in E10.5 chimeras. Arrow shows more ASCs were detected than ESCs in chimeras. Scale bar, 100 μm. **(E)** ASCs (tdTomato) contributed to mesonephros and gonad in E12.5 chimeras. Note, arrow indicates ASCs contributed to PGCs which were GOF-GFP positive cells. Scale bars, 100 μm. **(F)** A single cell from ASCs (GOF-GFP/tdTomato) contributed to chimeras at E6.5 to E10.5. White arrow indicates embryo; yellow arrow shows yolk sac; red arrow shows placenta. Scale bars in E6.5-7.5, 200 μm; scale bars in E9.5-10.5, 2 mm. **(G)** Multiple or single ESC-ASCs (GOF-GFP/tdTomato) contributed to embryo, yolk sac, and placenta in E10.5 chimeras. White arrow indicates embryo; yellow arrow shows yolk sac; red arrow shows placenta. Scale bars, 2 mm.
