# Supplementary material for: Derivation of hypermethylated pluripotent embryonic stem cells with high potency
Source: Cell Res. 2017 Oct 27;28(1):22–34. doi: 10.1038/cr.2017.134 (PMC5752839; doi:10.1038/cr.2017.134)
Supplement: Supplementary information, Figure S3 — RNAseq analysis of AFSCs and ASCs. [file cr2017134x3.pdf]

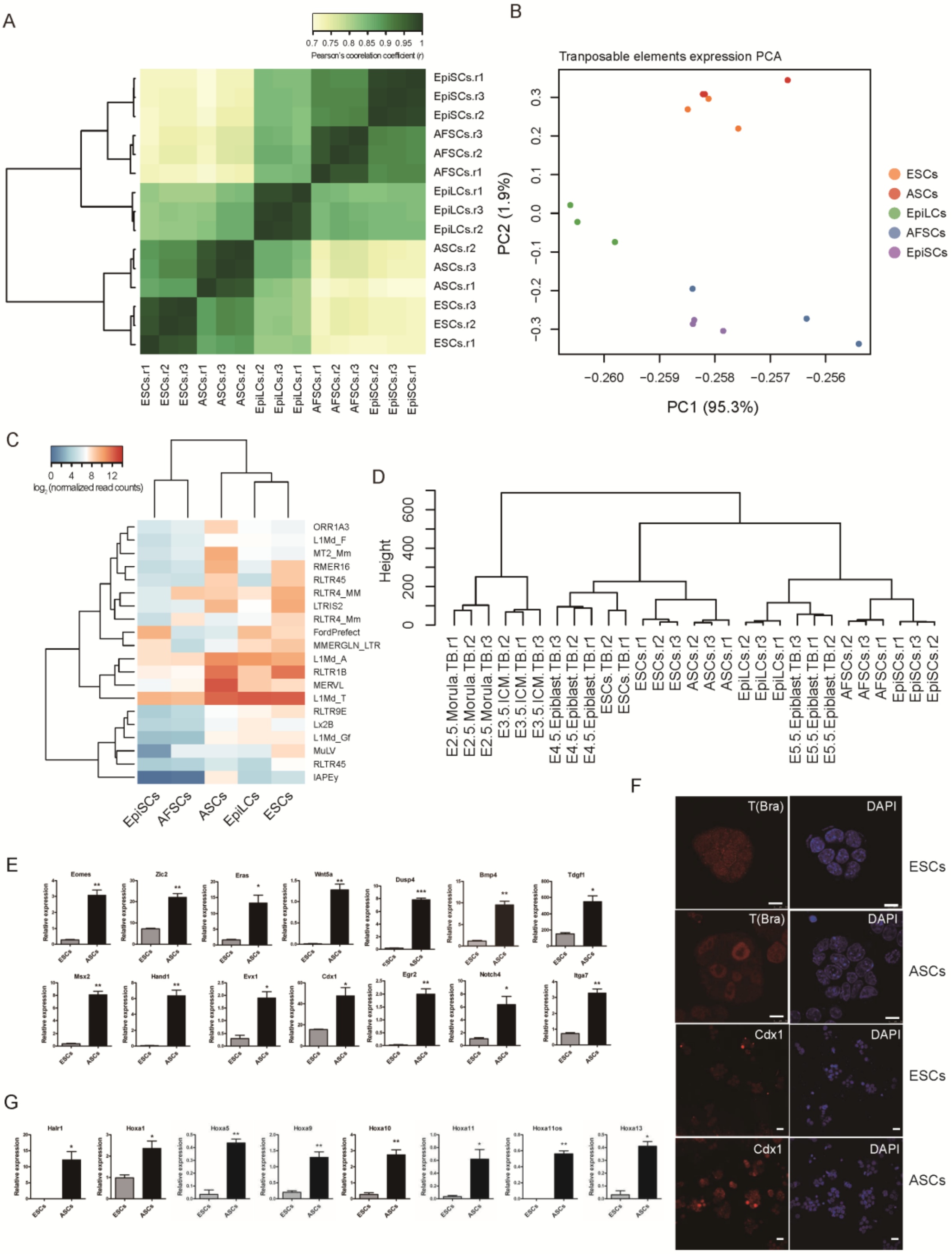

Supplementary information Figure 3 RNAseq analysis of AFSCs and ASCs.

(A) Heatmap showing Pearson correlation coefficient (*r*) of whole-genome transcriptome between samples and UHC with 1-*r* as a distance measure. **(B)** PCA of transposable element expression. **(C)** Heatmap showing expression of differentially expressed transposable elements. **(D)** UHC of gene expression of *in vitro* pluripotent stem cells and that of E2.5 to E5.5 embryos [37]. The analysis was based on 1 685 dynamically expressed genes between E2.5 to E5.5 embryos. **(E)** Mesoderm gene expression in ESCs and ASCs. **(F)** Immunofluorescence (IF) staining for T (Bra) and Cdx1 in ESCs and ASCs, 20 μm. **(G)** *Haunt/Halr1* and a set of *Hoxa* gene expression in ESCs and ASCs.
