# Supplementary material for: Derivation of hypermethylated pluripotent embryonic stem cells with high potency
Source: Cell Res. 2017 Oct 27;28(1):22–34. doi: 10.1038/cr.2017.134 (PMC5752839; doi:10.1038/cr.2017.134)
Supplement: Supplementary information, Figure S4 — 2-cell genes expression in ASCs. [file cr2017134x4.pdf]

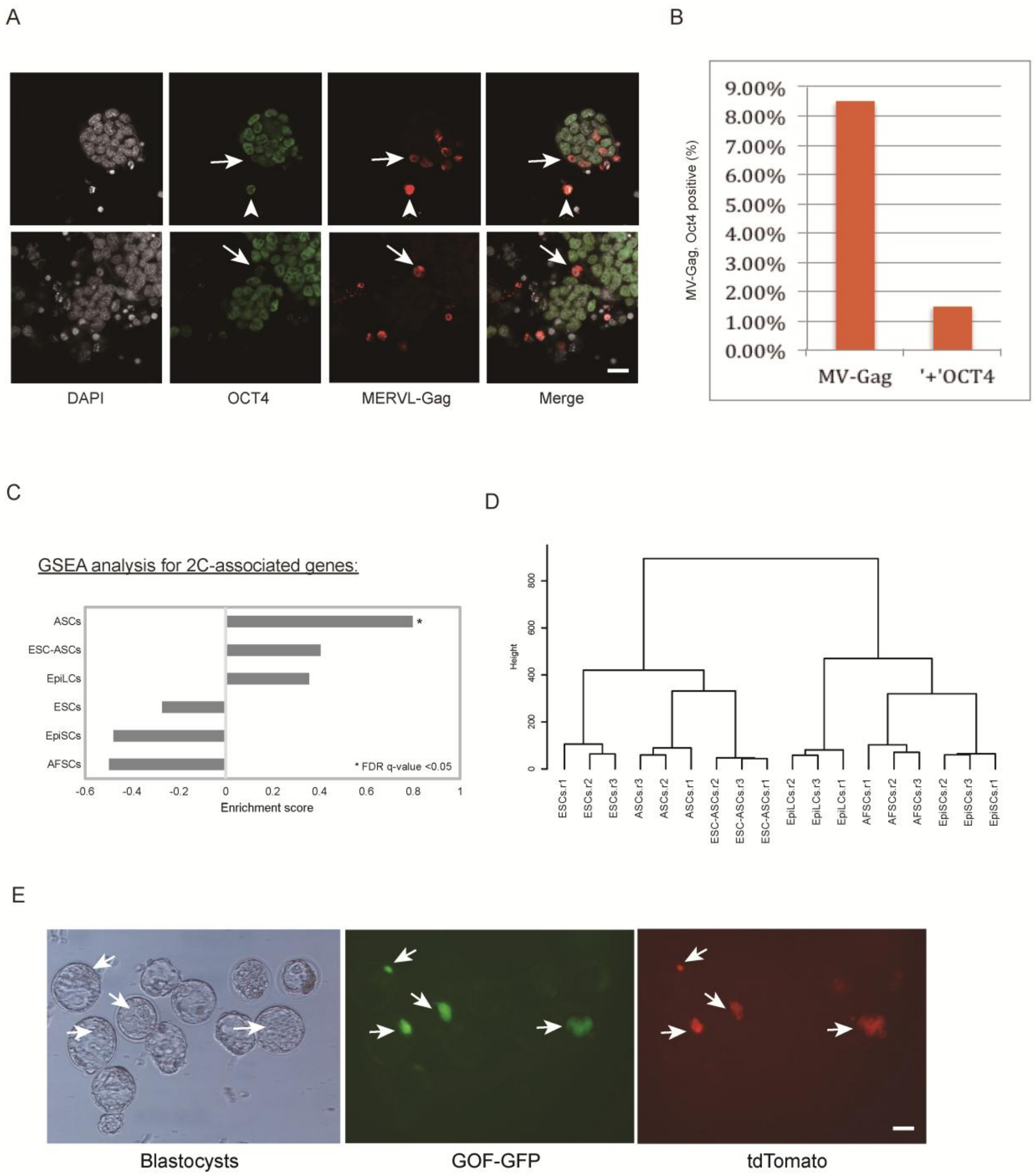

Supplementary information Figure 4 2-cell genes expression in ASCs.

(A) Immunofluorescence of MERVL-gag and OCT4 in ASCs, both rarely expressed (0.05%) in 2i/LIF ESCs. Arrows show MERVL-Gag-positive and OCT4 negative cells; arrowhead show cells positive for both MERVL-Gag and OCT4. Scale bars, 20μm. **(B)** Quantification of MERVL-Gag-positive (MV-Gag) and MERVL-Gag + Oct4-positive cells ('+'OCT4) amongst all cells. **(C)** Gene set enrichment analysis (GSEA) of 2C-associate genes [43]. Note that only ASCs were significantly enriched for 2C genes. **(D)** UHC of gene expression showing that ESC-ASCs cluster with ASCs. **(E)** Diagrams showing the injection of a single tdTomato/GOF-GFP ASC into 8-cell stage embryo, observed after culture 48 hours. Arrows show ASCs only exists in ICM. Scale bar, 50μm.
