# Supplementary material for: Derivation of hypermethylated pluripotent embryonic stem cells with high potency
Source: Cell Res. 2017 Oct 27;28(1):22–34. doi: 10.1038/cr.2017.134 (PMC5752839; doi:10.1038/cr.2017.134)
Supplement: Supplementary information, Figure S5 — Methylation landscape of ASCs. [file cr2017134x5.pdf]

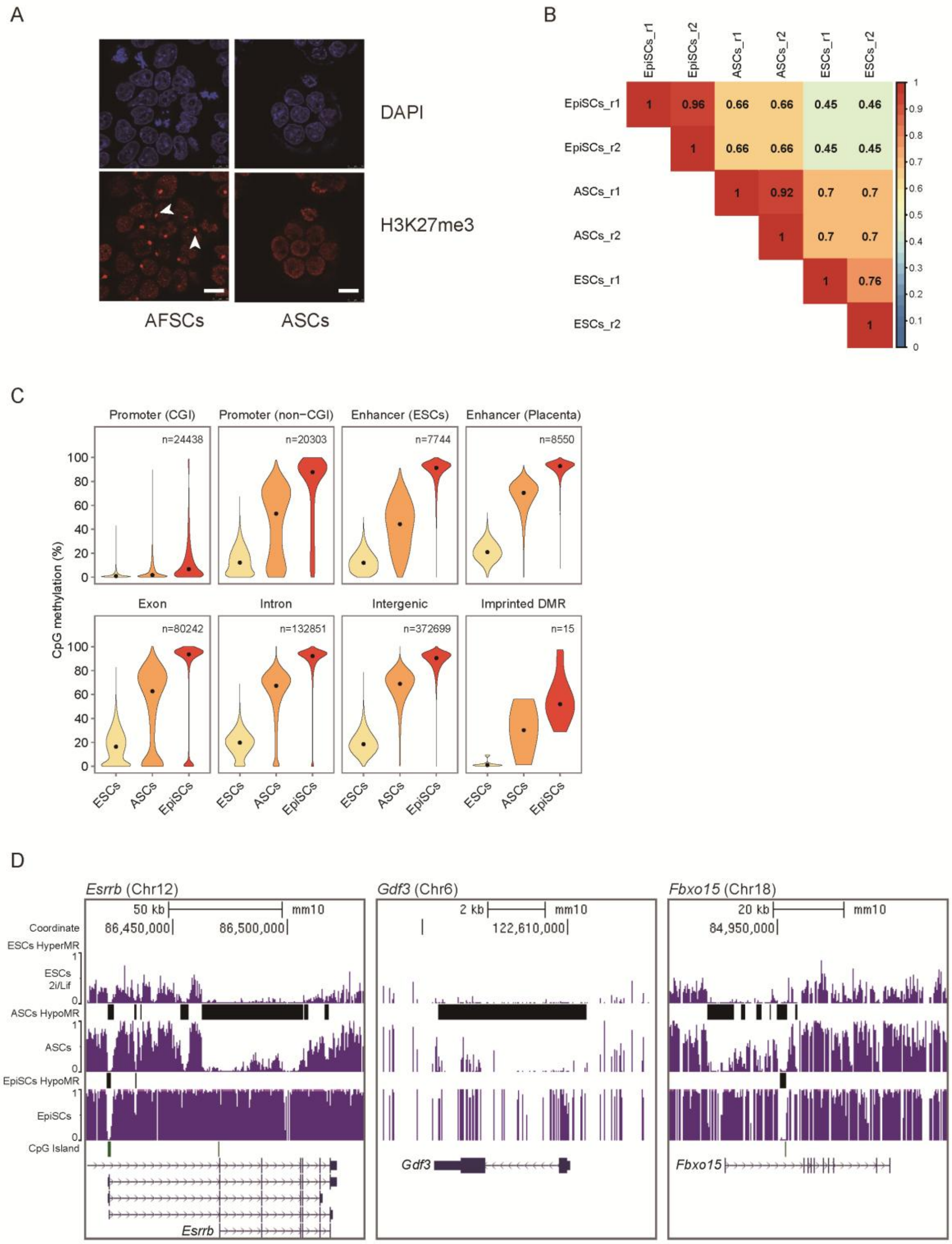

Supplementary information Figure 5 Methylation landscape of ASCs.

(A) X-chromosome inactivated in AFSCs, and reactivated in ASCs confirmed by lack of H3K27me3 spot. Arrowheads show X-chromosome inactivation with H3K27me3 spots in AFSCs. Scale bars, 20μm. **(B)** Correlation analysis of CpG methylation level of 2 kb genomic tiles between ESCs, ASCs and EpiSCs. Pearson correlation coefficient indicated. **(C)** Violin plot showing DNA methylation distribution at various genomic features. **(D)** UCSC genome browser snapshot of CpG methylation level at representative naive pluripotency genes.
