# Supplementary material for: Derivation of hypermethylated pluripotent embryonic stem cells with high potency
Source: Cell Res. 2017 Oct 27;28(1):22–34. doi: 10.1038/cr.2017.134 (PMC5752839; doi:10.1038/cr.2017.134)
Supplement: Supplementary information, Table S1 — The contribution of AFSCs and ASCs in chimera and tetraploid. [file cr2017134x6.pdf]

A

Stage of Embryo (collected)	Cell line (AFSCs)	No. of transfered embryos	No. of collected embryos	No. of LacZ positive embryos
**E6.5**	R1	32	28 (88%)	3 (11%)
**E6.5**	R4	20	14 (70%)	2 (14%)
**E7.5**	R4	100	89 (89%)	11 (12%)
**E8.5**	R4	42	22 (52%)	3 (14%)

B

kind of cell lines	Cell line	Derived ASCs	Repeat times
**G-3**	AFSCs	yes	6
**G-12**	AFSCs	yes	2
**G-6**	AFSCs	no	1
**G-4**	AFSCs	no	1
**ST2**	AFSCs	yes	1
**total**	AFSCs	3/5 (60%)	
**GOF-GFP**	EpiSCs	no	2
**x-GFP**	EpiSCs	yes	2
**ge-wild type**	EpiSCs	no	1
**stella-GFP**	EpiSCs	no	3
**total**	EpiSCs	1/4 (25%)	

C

Stage of Embryo (collected)	Cell line	No. of injected cell	No. of Transferred embryos	No. of collected embryos	No. of Gonad GPF+	No. of contribute body	No. of contribute yolk sack	No. of contribute placenta
**E12.5**	ASCs (Stella-GFP)	~15 cells	32	4 (13%)	3 (75%)	4 (100%)*	4 (100%)*	4 (100%)*
**E12.5**	ASCs (GOF-GFP)	~15 cells	37	6 (16%)	5 (83%)	6 (100%)*	6 (100%)*	6 (100%)*

* confirmed by Genomic PCR

D

Stage of Embryo	Cell line	No. of injected cell	No. of Transferred embryos	No. of pups	No. of chimera	No. of ♂,♀
**Full term**	ASCs	~15 cells	49	29 (59%)	6 (21%)	5, 1

E

Stage of Embryo (collected)	Cell line	No. of injected cell	No. of Transferred embryos	No. of collected embryos	No. of tdTomato positive	No. of Gonad GPF+	No. of contribute body	No. of contribute body+yolk sack	No. of contribute body+ yolk sac+placenta
**E10.5**	ASCs (GFP/Tomato)	~15 cells	20	6 (30%)	6 (100%)		6 (100%)	6 (100%)	5 (83%)
**E13.5**	ASCs (GFP/Tomato)	~15 cells	14	4 (29%)	4 (100%)	4 (100%)	4 (100%)	4 (100%)	2 (50%)

F

No. of recipients	Cell line	No. of injected cell	No. of Transferred embryos (2N+4N) left/right	No. of pups (2N+4N)	No. of 4N pups	No. of dead pups in 2 days	No. of 4N surviving pups	No. of ♂,♀
**No.1**	G-12 ASCs	~15 cells	5+7/5+7	5+5	5 (36%)	2 (4N)	3 (21%)	3, 0
**No.2**	G-12 ASCs	~15 cells	5+7/5+7	4+2	2 (14%)	0	2 (14%)	2, 0
**No.3**	G-12 ASCs	~15 cells	5+7/5+7	5+0	0	0	0	0
**No.4**	G-12 ASCs	~15 cells	5+7/5+7	0	0	0	0	0
**No.5**	G-12 ASCs	~15 cells	5+7/5+7	0	0	0	0	0
**Total**	G-12 ASCs	~15 cells	30/42	14+7	7/42(17%)	2(4N)	5/42(12%)	5,0

Supplementary information Table 1 The contribution of AFSCs and ASCs in chimera and tetraploid.

(A) Summary of chimeras derived from AFSCs R1 and R4 cell lines with Rosa26-lacZ reporter at E6.5-E8.5. **(B)** Derivation rate of ASCs. AFSCs cell lines are green, control EpiSCs are yellow. **(C)** Contribution of ASCs with GOF-GFP reporter and Stella-GFP reporter in E12.5 chimeras. **(D)** The number of full term chimeric pups derived from ASCs. **(E)** Contribution of ASCs (GOF-GFP/dtTomato) at E10.5 and E13.5. **(F)** The result of potency test by injecting ASCs to tetraploid embryos.
