# Supplementary material for: Derivation of hypermethylated pluripotent embryonic stem cells with high potency
Source: Cell Res. 2017 Oct 27;28(1):22–34. doi: 10.1038/cr.2017.134 (PMC5752839; doi:10.1038/cr.2017.134)
Supplement: Supplementary information, Table S2 — The summary of contribution of ASCs and ESC-ASCs in chimeras. [file cr2017134x7.pdf]

A

Stage of Embryo (collected)	Cell line	No. of injected cell	No. of transferred embryos	No. of collected embryos	No. of tdTomato positive	No. of contributed epiblast	No. of contributed VE	No. of contributed epiblast+EXE
E6.5	ASCs	single cell	62	36 (58%)	13 (36%)	13 (100%)	0	1 (7%)
E7.5	ASCs	single cell	58	29 (50%)	7 (24%)	7 (100%)	0	0

Stage of Embryo (collected)	Cell line	No. of injected cell	No. of transferred embryos	No. of collected embryos	No. of tdTomato positive	No. of contributed body	No. of contributed body+yolk sac	No. of contributed body+yolk sac+placenta
E8.5	ASCs	single cell	30	12 (40%)	6 (50%)	6 (100%)	4 (67%)	4 (67%)
E9.5	ASCs	single cell	30	15 (50%)	7 (47%)	7 (100%)	7 (100%)	5 (71%)
E10.5	ASCs	single cell	60	16 (27%)	5 (31%)	5 (100%)	5 (100%)	5 (100%)
E8.5-E10.5	ASCs	single cell	120	43 (36%)	18 (42%)	18 (100%)	16 (89%)	14 (78%)

B

Stage of Embryo (collected)	Cell line	No. of injected cell	No. of transferred embryos	No. of collected embryos	No. of tdTomato positive	No. of Gonad GPF+	No. of contributed body+yolk sac+placenta
E12.5	ASCs	single cell	36	13 (36%)	6 (46%)	6 (100%)	6 (100%)

C

Stage of Embryo	Cell line	No. of injected cell	No. of Transferred embryos	No. of pups	No. of chimera	No. of ♂,♀
Full term	ASCs	single cell	56	16 (29%)	7 (44%)	5, 2

D

Stage of Embryo (collected)	Cell line	No. of injected cell	No. of Transferred embryos	No. of collected embryos	No. of tdTomato positive	No. of contributed Epiblast	No. of contributed EXE	No. of contributed body	No. of contributed body+yolk	No. of contributed body+yolk sac +placenta
E6.5	SQ3.3-2i/LIF	~15 cells	20	10 (50%)	5 (50%)	5 (100%)	0			
E7.5	SQ3.3-2i/LIF	~15 cells	20	8 (40%)	3 (38%)	3 (100%)	0			
E10.5	SQ3.3-2i/LIF	~15 cells	44	23 (52%)	18 (78%)			18 (100%)	6 (33%)	1 (6%)
E10.5	SQ3.3-2i/LIF	single cell	45	20 (44%)	2 (10%)			2 (100%)	0	0

E

Stage of Embryo (collected)	Cell line	No. of injected cell	No. of transferred embryos	No. of collected embryos	No. of tdTomato positive	No. of contributed body	No. of contributed body+yolk	No. of contributed body+yolk sac+placenta
E10.5	E14/ESC-ASCs	~15 cells	34	22(65%)	11(50%)	11(100%)	11(100%)	10(91%)
E10.5	SQ3.3/ESC-ASCs	~15 cells	47	18(38%)	8(44%)	8(100%)	8(100%)	8(100%)
E10.5	SQ3.3/ESC-ASCs	single cell	17	15(88%)	6(40%)	6(100%)	6(100%)	6(100%)

Supplementary information Table 2 The summary of contribution of ASCs and ESC-ASCs in chimeras. **(A)** Contribution of single ASC (GOF-GFP/tdTomato) to chimeras at E6.5-E7.5 and E8.5-E10.5. **(B)** Contribution of single ASC (GOF-GFP/tdTomato) in E12.5 chimeras. **(C)** Full term chimeric pups were derived by single ASC. **(D)** Summary of chimeras derived from control ESCs, and collected at E6.5-E10.5. **(E)** Contribution of ESC-ASCs (GOF-GFP/tdTomato) in E10.5 chimeras.
